# Comparative efficacy of combination therapy including regenerative therapies versus monotherapy for erectile dysfunction: A systematic review and meta‐analysis

**DOI:** 10.1111/andr.70108

**Published:** 2025-08-12

**Authors:** Alberto Quistini, Giuseppe Fallara, Marco Tozzi, Massimiliano Depalma, Rocco Damiano, Alessandro Palmieri, Fabio Castiglione, Andrea Salonia, Roberto Bianchi, Matteo Ferro, Asif Muneer, Hussain M. Alnajjar, Karl H. Pang

**Affiliations:** ^1^ Department of Urology ASST Grande Ospedale Metropolitano Niguarda University of Milan Milan Italy; ^2^ Department of Urology ASST Santi Paolo e Carlo University of Milan Milan Italy; ^3^ Division of Urology IRCCS European Institute of Oncology University of Milan Milan Italy; ^4^ Urology Unit Magna Graecia University of Catanzaro Catanzaro Italy; ^5^ Department of Neurosciences Reproductive Sciences and Odontostomatology Urology Unit University of Naples “Federico II” Naples Italy; ^6^ School of Biomedical Engineering and Imaging Sciences King's College London London UK; ^7^ Vita‐Salute San Raffaele University Milan Italy; ^8^ Division of Experimental Oncology/Unit of Urology URI, IRCCS Ospedale San Raffaele Milan Italy; ^9^ Division of Surgery and Interventional Science University College London London UK; ^10^ Department of Urology University College London Hospitals NHS Trust London UK; ^11^ Department of Urology Chelsea and Westminster Hospital NHS Foundation Trust London UK

**Keywords:** erectile dysfunction, IIEF, Li‐ESWT, PDE5i

## Abstract

**Background and objective:**

The Current European Association of Urology guidelines do not provide recommendation for combination of regenerative therapies with standard therapies for erectile dysfunction. The aim of this study was to compare the efficacy of combined regenerative therapy with monotherapy for erectile dysfunction.

**Methods:**

A systematic review and meta‐analysis were conducted following the 2020 Preferred Reporting Items for Systematic Reviews and Meta Analyses guidelines. The protocol was registered on PROSPERO (CRD42024522307). Randomized controlled trials and prospective/retrospective studies comparing combination therapies (low‐intensity external shockwave therapy, platelet‐rich plasma, stem cell therapy with phosphodiesterase‐5 inhibitors, or other treatments) with monotherapy were included. Erectile function was assessed using the International Index of Erectile Function‐5 and Erection Hardness Scale. Groups were compared using standardized mean difference. Subgroup analyses based on treatment type, erectile dysfunction cause, and follow‐up duration were also conducted. Risk of bias was assessed using risk of bias 2 and Robins‐I tools.

**Key findings and limitations:**

Of 1416 articles screened, eight studies involving 553 patients met the inclusion criteria. All studies included phosphodiesterase‐5 inhibitors and low‐intensity external shockwave therapy as regenerative treatment, with no studies on stem cell therapy or platelet‐rich plasma. After treatment, no significant difference in International Index of Erectile Function scores was found between combination and monotherapy groups. However, subgroup analysis revealed that combination therapy showed a statistically significant improvement compared to low‐intensity external shockwave therapy alone (standardized mean difference: 0.61; 95% confidence interval: 0.13‒1.09; *p* = 0.013). A statistically significant improvement was found in vasculogenic (standardized mean difference: 0.65; *p* < 0.001) and diabetic cases (standardized mean difference: 1.05; *p* < 0.001).

**Conclusions and clinical implications:**

Combination of phosphodiesterase‐5 inhibitors and intensity external shockwave therapy resulted in significant improvement of International Index of Erectile Function compared to intensity external shockwave therapy alone. However, the risk of bias was high because of the low quality of the studies.

**Patient summary:**

Combination of phosphodiesterase‐5 inhibitor with intensity external shockwave therapy significantly improved erectile function, especially in patients with vasculogenic erectile dysfunction or diabetes.

## 1 INTRODUCTION

Erectile dysfunction (ED) is a common condition that refers to the inability to obtain and maintain sufficient penile erection for sexual intercourse.^1^

Until recently the treatment of this condition has mainly been based on a symptomatic approach, that is, a transient enhancement of penile erection, and not addressing ED causes, such as reversing endothelial dysfunction or restoring disrupted penile tissue homeostasis.^2^

The updated European Association of Urology (EAU) guidelines abandoned the traditional three‐tier concept and embraced a personalized approach based on ED causes, therapeutic availabilities, and patients’ expectations. Phosphodiesterase type‐5 inhibitors (PDE5i) still represent the first‐line treatment for ED, ideally alongside lifestyle modifications, in most cases.^3^, ^4^ Alternative therapies include intracavernous injections, vacuum device use, and topical alprostadil.^5^, ^6^, ^7^ If these options fail, penile prosthesis implantation is an option.^8^

During the last decades, new therapeutical modalities have been introduced in the treatment landscape of ED. Among these, regenerative therapies such as low‐intensity external shockwave therapy (Li‐ESWT), platelet‐rich plasma (PRP) injections, and stem cell therapy (SCT) are gaining interest.^12^, ^13^, ^14^ These treatment modalities seemed to potentially improve erectile function through biological mechanism such as vascular circulation improvement, stem cell activation, reduction of inflammation by down‐regulation of pro‐inflammatory cytokines, decrease of cavernosal fibrosis, and restoration of smooth muscle and supporting nerves.^11^

Limited research has explored the effects of combining conventional therapies for ED with these new regenerative therapies. Currently, there is a gap in data regarding whether the quality of erections may improve when comparing a monotherapy— conventional or regenerative—with a combination therapy that includes at least one regenerative treatment modality.

The objective of this review is to determine the benefit of combining at least one regenerative therapy for ED, that is, Li‐ESWT, PRP, and SCT with PDE5i or other conventional ED therapies, compared to using monotherapy for ED. The hypothesis tested is whether regenerative therapy should be better used alone or in combination with other ED treatments.

## 2 EVIDENCE ACQUISITION

### 2.1 Search strategies and selection criteria

We conducted a systematic review and metanalysis of all published randomized controlled trials (RCTs), prospective and retrospective interventional studies, assessing the effect of combination therapy versus monotherapy (in the form of oral PDE5i, intracavernosal injections, topical or intraurethral alprostadil, vacuum device, Li‐ESWT, SCT, and PRP) on erectile function. The review was conducted according to the Preferred Reporting Items for Systematic Reviews and Meta Analyses (PRISMA) 2020 guidelines.^12^ Medline/PubMed, Google Scholar, Cochrane Library, and Scopus databases were queried for studies indexed up to March 5, 2024. The protocol was registered in PROSPERO (CRD42024522307).

Combinations of the following keywords were used for the search included: “erectile dysfunction,” “combination therapy,” “stem cell,” “oral phosphodiesterase type 5 inhibitors,” “intracavernosal injections,” “alprostadil,” “vacuum device,” “low‐intensity extracorporeal shockwave therapy,” and “platelet‐rich plasma.” Review articles, commentaries, editorials, and articles that did not undergo peer‐review were excluded. Reference lists of included studies were hand‐searched for completeness. Titles and abstracts of manuscripts were used to screen for initial study inclusion. Full text review was performed when the abstract was not sufficient to determine study inclusion. Inclusion and exclusion criteria were based on the PICOS criteria: population—male subjects with ED aged >18 years; intervention—combination therapy including two medical treatments, which encompasses at least one regenerative therapy modality, that is, PRP, SCT or Li‐ESWT and conventional therapies; comparison—monotherapy, including oral PDE5i, intracavernosal injections, topical or intraurethral alprostadil, vacuum device, PRP, SCT, and low‐intensity shockwave treatment (Li‐SWT); outcomes—erectile function, as defined according to validated questionnaires, that is, the International Index of Erectile Function‐5 (IIEF‐5) and Erection Hardness Scale (EHS); studies—RCTs or prospective non‐randomized comparative interventional studies or retrospective interventional studies.

Two authors completed the study selection independently (A.Q. and G.F.). Potential disagreements were resolved by consensus among all co‐authors.

### 2.2 Variables and outcomes definition

Data were collected according to a proforma, which included year of publication, year of enrolment, authors names, study design, ED cause and duration, number of men stratified according to treatment modality, type of regenerative treatment, type of conventional treatment, regenerative treatment duration and details regarding dose and timing of the treatment, and erectile function measured with validated questionnaires (IIEF‐5 and EHS) at baseline and at the end of follow‐up.

The primary outcome was to assess a difference in terms of erectile function between the combination therapy and monotherapy groups at the end of follow‐up.

### 2.3 Statistical analysis

Whereas the studies provided the median (interquartile range) and not the mean and standard deviation (SD) of the IIEF and EHS scores, these were mathematically transformed to mean ± SD, according to the Box‒Cox method.^13^ The standardized mean difference (SMD) was used to compare IIEF and EHS scores in combination versus monotherapy groups at the end of follow‐up. The SMD was used to compare IIEF and EHS scores at baseline and at the end of follow‐up for each group, that is, the combination and the monotherapy group. A subgroup analysis was conducted according to the modality treatment in the control arm, that is, regenerative therapy, PDE5i, or other non‐regenerative therapy for ED.

In addition, in order to account for possible heterogeneity related to the indication for ED treatment, a subgroup analysis was conducted according to the causes of the ED, that is, vasculogenic, diabetes, mixed, and post‐radical prostatectomy. Moreover, to account for the different follow‐up durations across the studies, a subgroup analysis was conducted based on the length of follow‐up at 3, 6, or 12 months when data were available, or, in the absence of these specific time points, at the end of the follow‐up period as reported in each study. The interstudy heterogeneity was evaluated using the *I* ^2^ and tau statistics. To estimate the possible publication bias, funnel plots were used for each outcome.

The risk of bias (RoB) was determined using the RoB tool (RoB 2) for RCTs and the RoB in non‐randomized studies of interventions (Robins‐I). Any disagreement was resolved with consensus or by a third reviewer.

Statistical analyses were performed using the RStudio graphical interface v.0.98 for R software environment v.3.0.2.

## 3 EVIDENCE SYNTHESIS

### 3.1 Studies overview

**FIGURE 1:**
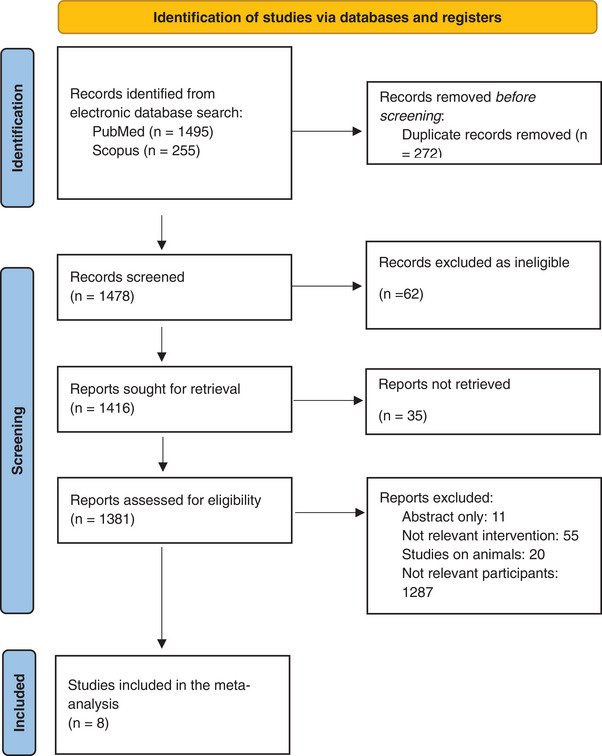
Preferred Reporting Items for Systematic Reviews and Meta Analyses (PRISMA) flowchart for study selection.

Of 1416 articles identified, eight were eligible for inclusion, involving 553 patients (Figure 1). Table 1 summarizes the characteristics of the included studies. The oldest study was published in 2016^14^ and the latest in 2022.^15^, ^16^, ^17^, ^18^, ^19^ Seven studies were prospective whereas one was retrospective. Four studies enrolled patients with vasculogenic ED,^14^, ^16^, ^18^, ^19^ one study enrolled patients with ED caused by diabetes mellitus,^20^ two studies enrolled patients with ED as a consequence of radical prostatectomy,^17^, ^21^ whereas one study enrolled patients with mixed cause ED.^15^ The duration of ED before the enrolment in the study ranged between 7^17^ and 48^18^ months, whereas it was not available in four studies.^14^, ^16^, ^20^, ^21^ In four studies,^14^, ^16^, ^18^, ^21^ patients were on PDE5i treatment and were required to discontinue it around a month before being enrolled in the screening, in three studies these data were not available whereas in one study^19^ PDE5i assumption before screening was an exclusion criterion.

**TABLE 1:** Characteristics of all included studies.

Author and year of publication	Title	Study design	Number of participants (combination arm)	Number of participants (single arm)	ED duration (months)	ED cause	PDE5i naïve	Combination therapy versus monotherapy
Gallo et al., 2022^16^	Adjuvant daily therapy with L‐arginine 2500 mg and tadalafil 5 mg increases efficacy and duration of benefits of low‐intensity extracorporeal shockwave therapy for ED: a prospective, randomized, single‐blinded study with 1‐year follow‐up	Prospective randomized trial	41	42	NA	Vasculogenic	No	Li‐SWT + PDE5i versus Li‐SWT
Motil et al., 2022^17^	Linear low‐intensity extracorporeal shockwave therapy as a method for penile rehabilitation in ED patients after radical prostatectomy: a randomized, single‐blinded, sham‐controlled clinical trial	Prospective randomized trial	16	16	7	Post‐RP	Yes	Li‐SWT + PDE5i versus PDE5i or PGE1
Baccaglini et al., 2020^21^	Role of the low‐intensity extracorporeal shockwave therapy on penile rehabilitation after radical prostatectomy: a randomized clinical trial	Prospective randomized trial	36	41	NA	Post‐RP	Both	Li‐SWT + PDE5i versus PDE5i
Sandoval‐Salinas et al., 2022^15^	Are radial pressure waves effective for the treatment of moderate or mild to moderate ED? A randomized sham therapy controlled clinical trial	Prospective randomized trial	37	39	>3	Mixed causes	Yes	Li‐SWT + PDE5i versus PDE5i
Verze et al., 2020^20^	Efficacy and safety of Li‐SWT plus tadalafil 5 mg once daily in men with type 2 DM and ED: a matched‐pair comparison study	Retrospective	78	78	NA	DM	No	Li‐SWT + PDE5i versus PDE5i
Mykoniatis et al., 2022^18^	Effect of combination treatment with Li‐SWT and tadalafil on mild and mild‐to‐moderate ED: a double‐blind, randomized, placebo‐controlled clinical trial	Prospective randomized trial	25	25	48	Vasculogenic	Yes	Li‐SWT + PDE5i versus Li‐SWT
Kitrey et al., 2016^14^	Penile Li‐SWT is able to shift PDE5i non‐responders to responders: a double‐blind, sham‐controlled study	Prospective randomized trial	37	16	NA	Vasculogenic	Yes	Li‐SWT + PDE5i versus PDE5i
Setiawan et al., 2022^19^	An update in improving ED therapy in Indonesia by using Li‐ESWT and tadalafil combination—vascular endothelial growth factor and peak systolic velocity comparison: a randomized clinical trial	Prospective randomized trial	14	12	31.2	Vasculogenic	No	Li‐SWT + PDE5i versus PDE5i

Abbreviations: DM, diabetes mellitus; ED, erectile dysfunction; IIEF‐5, International Index of Erectile Function Questionnaire‐5; Li‐ESWT, low‐intensity external shockwave therapy; Li‐SWT, low‐intensity shockwave treatment; NA, not available; PDE5i, phosphodiesterase type 5 inhibitors; PGE1, prostaglandin E1; RP, radical prostatectomy.

The combination therapy consisted of Li‐ESWT plus PDE5i in all the studies considered (321 patients overall). The amount of shockwaves delivered during each Li‐ESWT cycle and the period of treatment varied across all the included studies (Table S1). Monotherapy consisted of PDE5i in five studies^14^, ^15^, ^19^, ^20^, ^21^ (186 patients overall); Li‐ESWT in two studies^16^, ^18^ (67 patients overall); and either PDE5i or prostaglandin E1 therapy in one study^17^ (16 patients overall). None of the selected studies included patients who were treated with SCT or PRP.

All the included studies measured the erectile function with the IIEF‐5 and three studies used the EHS as well.^15^, ^16^, ^19^

Heterogeneity of the effect size across the included studies was high, with *I* ^2^ ranging between 75% and 97% for every outcome. According to the RoB 2 (Table S2A,B) and Robins‐I tools (Table S3), the RoB was considered high or serious across all the included studies.

### 3.2 Erectile function at baseline

At baseline, there was no statistically significant difference in the IIEF score between the combination group and the monotherapy group (SMD: ‒0.04; 95% confidence interval [CI]: ‒0.21 to 0.13; *p* = 0.6) (Figure S1).

### 3.3 Erectile function at end of follow‐up

**FIGURE 2:**
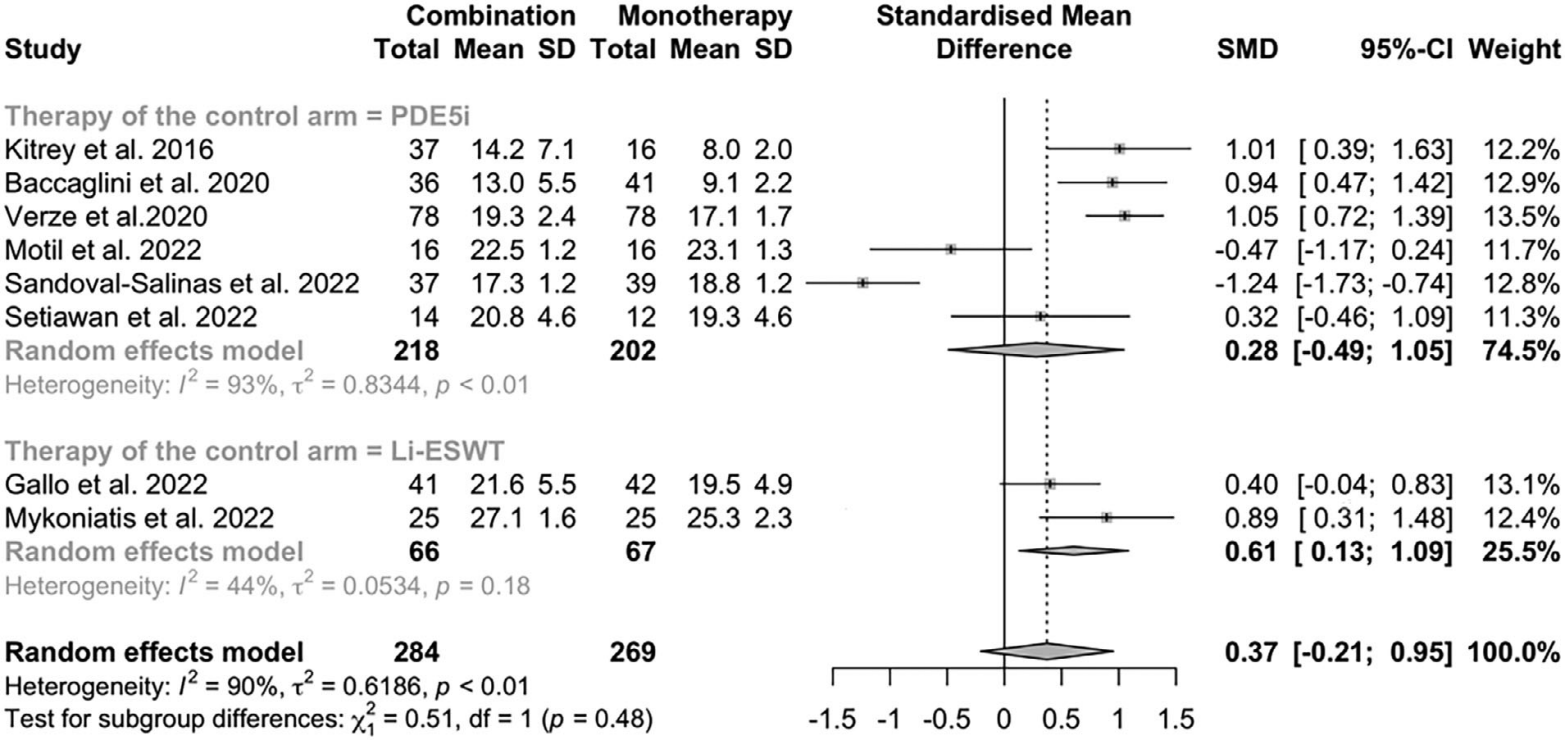
Forest plot of post‐treatment International Index of Erectile Function Questionnaire‐5 (IIEF‐5) scores in the combination versus monotherapy groups, stratified by the type of monotherapy (phosphodiesterase‐5 inhibitors [PDE5i] or low‐intensity external shockwave therapy [Li‐ESWT]). SD, standard deviation; SMD, standardized mean difference; 95% CI, 95% confidence interval.

Considering all the studies, no statistically significant difference in the IIEF scores (SMD: 0.37; 95% CI: ‒0.21 to 0.95; *p* = 0.3) between the combination and the monotherapy group was found post‐treatment (Figure 2). However, in the subgroup analysis, a statistically significant difference was found in the post‐treatment IIEF score (SMD: 0.61; 95% CI: 0.13‒1.09; *p* = 0.013) comparing combination therapy versus Li‐ESWT monotherapy. In contrast, no statistically significant difference was found when the monotherapy consisted of PDE5i alone (*p* = 0.5). No statistically significant difference in the EHS scores between the combination therapy group and the monotherapy group was found post‐treatment, in both the overall and the subgroup analysis (*p* > 0.05) (Figure S2).

Given the different lengths of follow‐up duration, a supplementary analysis including only studies with 6 months follow‐up was performed (Figure S3). A statistically significant difference in the IIEF was found when comparing combination with monotherapy in the overall analysis (SMD: 0.58; 95% CI: 0.05‒1.12; *p* = 0.033) and in the subgroup of studies where monotherapy consisted in Li‐ESWT (SMD: 0.60; 95% CI: 0.12‒1.09; *p* = 0.015).

**FIGURE 3:**
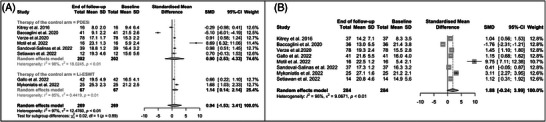
Forest plot of the difference in post‐treatment International Index of Erectile Function Questionnaire‐5 (IIEF‐5) scores compared to baseline in the monotherapy (A) and combination (B) groups. Li‐ESWT, low‐intensity external shockwave therapy; PDE5i, phosphodiesterase type 5 inhibitors; SD, standard deviation; SMD, standardized mean difference; 95% CI, 95% confidence interval.

We also assessed erectile function post‐treatment within the same group. There was no statistically significant difference in the post‐treatment IIEF scores compared to baselines in the case of monotherapy (SMD: 0.94; 95% CI: ‒1.53 to 3.41; *p* = 0.5); however, a statistically significant difference was found when considering only those studies where the Li‐ESWT was used (SMD: 1.14; 95% CI: 0.14‒2.14; *p* = 0.026) and not when considering studies on PDE5i (*p* = 0.2) (Figure 3A). In addition, there was no statistically significant difference in the post‐treatment IIEF scores compared to baselines in the case of combination therapy (SMD: 1.88; 95% CI: ‒0.24 to 3.99; *p* = 0.08) (Figure 3B). At supplementary analysis after excluding the study of Baccaglini et al.^21^ because it reported pre‐prostatectomy IIEF scores as baseline (Figure S4), there was a statistically significative difference in the post‐treatment overall score compared to baseline in the combination group (SMD: 2.39; 95% CI: 0.26‒4.52; *p* = 0.04), whereas no differences were found in the monotherapy group. EHS results are shown in Figure S5, but no statistically significant difference was found comparing post‐treatment with baseline scores.

**FIGURE 4:**
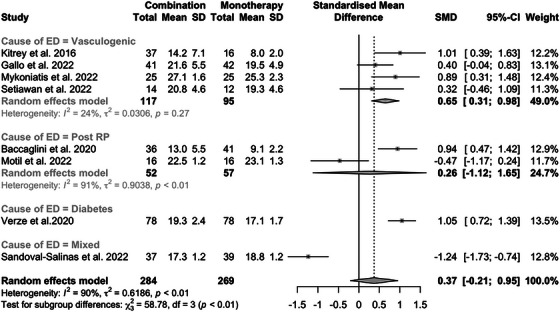
Forest plot of post‐treatment International Index of Erectile Function Questionnaire‐5 (IIEF‐5) scores in the combination versus monotherapy groups, stratified by causes of erectile dysfunction (ED). RP, radical prostatectomy; SD, standard deviation; SMD, standardized mean difference; 95% CI, 95% confidence interval.

When the post‐treatment IIEF scores in the combination versus monotherapy groups were evaluated and stratified by causes of ED, a statistically significant difference emerged only in the cases of vasculogenic and diabetes‐related ED (SMD: 0.65; 95% CI: 0.3‒0.98; *p* < 0.001 and SMD: 1.05; 95% CI: 0.72‒1.39; *p* < 0.001, respectively) (Figure 4).

### 3.4 Publication bias

Funnel plots showed a modest publication bias across all analyzed outcomes (Figure S6).

## 4 DISCUSSION

In this systematic review and meta‐analysis we found that Li‐ESWT in association with PDE5i might increase IIEF scores more than Li‐ESWT alone. This works better in the case of vasculogenic ED. The RoB highlights important limitations of the current literature on the issue, providing an overall low grade of evidence. No studies on PRP or SCT combination were found. This finding elicits some important consideration regarding Li‐ESWT protocols and indications.

The last version of the EAU guidelines provided a comprehensive therapeutic and decision‐making algorithm to assist clinicians in navigating the current complexities of ED treatment. Alongside lifestyle modifications, PDE5i continue to serve as a cornerstone of ED management.^3^, ^4^, ^22^, ^23^ However, a significant proportion of patients, over 50%, reported dissatisfaction, low adherence, or discontinuation of conventional therapies because of insufficient efficacy, adverse events, or contraindications.^24^

Before considering penile prosthesis implantation, some studies recommend exploring combination therapies, as they may offer additional benefits.^25^ In 2021, Mykoniatis et al. demonstrated that combining PDE5i with antioxidants, tadalafil, Li‐ESWT, or vacuum devices could improve outcomes for ED patients who are unresponsive to PDE5i alone, without any significant increase in adverse events.^26^

Over the past decades, new therapeutic modalities have been introduced for the treatment of ED, addressing its pathophysiologic mechanism.^9^ In this context, Li‐ESWT, PRP, and SCT are examples of therapies that aim to improve erectile function by addressing various pathogenetic mechanisms, including enhancing vascular circulation, activating stem cells, supporting nerve regeneration, reducing cavernosal fibrosis, and restoring smooth muscle content.^10^, ^15^ The standalone efficacy of these therapies for ED remains less clear. While some studies have reported improvements in penile hemodynamic parameters following Li‐ESWT, the clinical significance of these findings remains uncertain.^27^ Additionally, some RCTs suggested that better outcomes may be achieved by combining Li‐SWT with other treatments, such as daily tadalafil.^18^ However, the EAU Guidelines have yet to provide a definitive position on whether Li‐SWT should be used alone or in combination with PDE5i.

The hypothesis behind combining PDE5i with regenerative therapies is based on their potential to synergistically enhance the restoration of erectile function by boosting tissue regeneration and optimizing the vascular environment.^28^ Li‐ESWT has been shown to stimulate the release of angiogenic factors, including vascular endothelial growth factor, endothelial nitric oxide synthase, and proliferating cell nuclear antigen, promoting tissue regeneration, neo angiogenesis, and improved local blood supply.^11^ These mechanisms also address peripheral neuropathy, endothelial dysfunction, and pathological fibromuscular alterations in the corpus cavernosum.^21^ Additionally, Li‐ESWT enhances cyclic guanosine monophosphate (cGMP) synthesis, a messenger critical for smooth muscle relaxation during erection, further supporting the hypothesis that its combination with PDE5i may achieve superior outcomes.^11^ The vacuum therapy has also been shown to preserve penile size and improve arterial blood inflow, creating favorable conditions for Li‐ESWT application.^29^, ^33^

The results of this systematic review and meta‐analysis further support these hypotheses but are not devoid of limitations. First, although no statistically significant difference in baseline erectile function was observed between the combination and monotherapy groups ensuring their comparability at least regarding their baseline erectile function, the population examined in our study was heterogeneous. Regarding the ED duration, this was not consistently reported across the studies included in this reviewed. According to Cho et al., the duration of ED is a significant risk factor for the development of cavernous tissue fibrosis in a rat model. Thereof, it is reasonable to consider that the longer the ED persists, the higher the likelihood that fibrotic changes in the cavernous tissue will occur, potentially complicating treatments outcomes and worsening the condition.^30^ This fact has been confirmed by Adeldaeim et al., who concluded that the success of Li‐ESWT occurred especially in young patients with mild‐moderate ED, short duration of ED and no associated comorbidities.^31^

Furthermore, many studies lacked detailed information on prior ED treatments, making it difficult to assess the impact of previous interventions. The study population exhibited variability in the ED therapies used prior to enrolment. Some patients had discontinued their treatments weeks or months before inclusion^16^, ^18^ while others continued therapy.^18^, ^24^ Furthermore, the population included both partial responders to treatment^18^, ^21^ and those who were refractory,^14^ thus introducing additional variability to the patient profiles. Few RCTs have been conducted on this specific topic of comparing combination therapy versus monotherapy for ED, and those included in the metanalysis did not often specify the overmentioned factors, like prior use of PDE5i, whether patients were responders or refractory to such treatments and ED duration previous treatment. Thus, in order to improve the homogeneity of the study population and better assess treatment outcomes, future RCTs should consider specifying such criteria of inclusion and exclusion.

A significant variation across studies in the Li‐SWT protocols, including differences in the number of weekly applications, total treatment duration, energy delivered per session, session length, and the inclusion of break periods between cycles was also found. Hayon et al. highlighted that the global application of Li‐ESWT for ED is limited by the lack of standardized treatment protocols, differing patient populations, and variable outcome measurements. To standardize the approach, they reviewed randomized, sham‐controlled trials published over the last 10 years and found that, despite protocol variations, common settings included an energy level of 0.09 mJ/mm^2^, a frequency of 5 Hz, and 1500 shocks per session. They also noted variations in treatment sessions (4‒12 sessions) and intervals. Despite these differences, the studies generally showed positive treatment effects, emphasizing the need for consensus on an optimal protocol and the challenges of comparing studies with such variability.^32^

In addition, the follow‐up duration varied across studies, which may affect the observed outcomes and their long‐term stability. A sensitivity analysis was conducted analysing the outcomes at 6 months follow‐up date. Many studies have evaluated the needing for establish the long‐term efficacy of Li‐ESWT for ED; a review by Brunckhorst et al. concluded that Li‐ESWT has shown significant benefits for ED at 6 months, with a median IIEF score improvement of 5.3 points. However, studies with follow‐up beyond 6 months showed limited further improvement, with studies that reported a plateau in results, while others indicated a gradual decline in effectiveness. These findings highlight the need for extended follow‐up periods to better assess the long‐term efficacy of Li‐ESWT and to standardize study protocols for more reliable conclusions.^33^

Moreover, not all studies were prospective in their design, and the included RCT were evaluated as being at high RoB providing low level of evidence. In general, the studies were small, there were concerns regarding the randomizations processes and the blinding of investigators and participants to the intervention or control arms. Of note, the study by Verze et al.^20^ was a retrospective analysis of a prospectively maintained database, which adds further heterogeneity to the results. Finally, certain regenerative therapies, such as PRP and SCT, were excluded from our review because of the absence of comparative studies investigating their use in combination therapies within our systematic search. However, preliminary data suggest that incorporating PDE5i into PRP or SCT protocols may provide additional benefits.^2^, ^34^, ^35^, ^36^

## 5 CONCLUSIONS

This study evaluated the efficacy of combination therapies‐ i.e., a regenerative treatment combined with a conventional therapy‐ versus monotherapy for erectile dysfunction. The combination of phosphodiesterase‐5 inhibitors with low‐intensity external shockwave therapy resulted in significant improvements in erectile function, suggesting that this combination may be particularly beneficial for patients with vasculogenic or diabetic erectile dysfunction, who often have limited response to phosphodiesterase‐5 inhibitors alone.

Unfortunately, data on other regenerative therapies such as platelet‐rich plasma and stem cell therapy were not available. Further research is needed to confirm the long‐term benefits of combination therapies and to identify the patient subgroups most likely to benefit from these treatments.

## Supporting information



Supporting Information

Supporting Information
