# Supplementary material for: Comparative efficacy of combination therapy including regenerative therapies versus monotherapy for erectile dysfunction: A systematic review and meta‐analysis
Source: Andrology. 2025 Aug 12;14(2):358–67. doi: 10.1111/andr.70108 (PMC12842854; doi:10.1111/andr.70108)
Supplement: Supplementary file 1 — Supporting Information [file ANDR-14-358-s001.docx]

| Supplementary Table 1. Characteristics of the Li-ESWT treatment in all included studies | | |
| --- | --- | --- |
| Author; year of publication | **Title** | **Schedule** |
| Gallo et al. 2022 | Adjuvant daily therapy with L-arginine 2,500 mg and tadalafil 5 mg increases efficacy and duration of benefits of low-intensity extracorporeal shock wave therapy for erectile dysfunction: A prospective, randomized, single-blinded study with 1-year follow-up | 1 weekly application for 6 weeks. Each session consisted of 3000 SW |
| Motil et al. 2022 | Linear Low-Intensity Extracorporeal Shockwave Therapy as a Method for Penile Rehabilitation in Erectile Dysfunction Patients after Radical Prostatectomy: A Randomized, Single-Blinded, Sham-Controlled Clinical Trial. | 1 weekly application for 4 weeks. Each session consisted of 4000 SW |
| Baccaglini et al. 2020 | The Role of the Low-Intensity Extracorporeal Shockwave Therapy on Penile Rehabilitation After Radical Prostatectomy: A Randomized Clinical Trial | 1 weekly application for 8 weeks. |
| Sandoval-salinas et al. 2022 | Are Radial Pressure Waves Effective for the Treatment of Moderate or Mild to Moderate Erectile Dysfunction? A Randomized Sham Therapy Controlled Clinical Trial | 1 weekly application for 6 weeks. Each session consisted of 4000 SW in the body of the penis and 2000 SW the perineal area. |
| Verze et al. 2020 | Efficacy and safety of low-intensity shockwave therapy plus tadalafil 5 mg once daily in men with type 2 diabetes mellitus and erectile dysfunction: a matched-pair comparison study | 2 weekly applications for 3 weeks. Each session consisted of 1500 to 2400 SW. |
| Mykoniatis et al. 2022 | The Effect of Combination Treatment With Low-Intensity Shockwave Therapy and Tadalafil on Mild and Mild-To-Moderate Erectile Dysfunction: A Double-Blind, Randomized, Placebo-Controlled Clinical Trial | 2 weekly applications for 3 weeks. Each session consisted of 5000 SW. |
| Kitrey et al. 2016 | Penile Low Intensity Shock Wave Treatment is Able to Shift PDE5i Non responders to Responders: A Double-Blind, Sham Controlled Study | 2 weekly applications for 3 weeks. Each session consisted of 1500 SW. |
| Setiawan et al. 2022 | An update in improving erectile dysfunction therapy in Indonesia by using Li-ESWT and tadalafil combination — vascular endothelial growth factor and peak systolic velocity comparison: a randomized clinical trial | 2 weekly applications for 4 weeks. Each session consisted of 1500 SW. |

*Legend* – SW: Shock waves; Li-ESWT: Low Intensity External Shock Wave Therapy

**Supplementary Table 2A**. Risk of bias for the study regarding the erectile function measured with IIEF-5, at baseline vs at the end of follow up, according to the RoB-II tool

| Author | Title | D1 | D2 | D3 | D4 | D5 | Overall |
| --- | --- | --- | --- | --- | --- | --- | --- |
| Baccaglini et al. | The Role of the Low-Intensity Extracorporeal Shockwave Therapy on Penile Rehabilitation After Radical Prostatectomy: A Randomized Clinical Trial |  |  |  |  |  |  |
| Gallo et al. | Adjuvant daily therapy with L-arginine 2,500 mg and tadalafil 5 mg increases efficacy and duration of benefits of low-intensity extracorporeal shock wave therapy for erectile dysfunction: A prospective, randomized, single-blinded study with 1-year follow-up |  |  |  |  |  |  |
| Motil et al. | Linear Low-Intensity Extracorporeal Shockwave Therapy as a Method for Penile Rehabilitation in Erectile Dysfunction Patients after Radical Prostatectomy: A Randomized, Single-Blinded, Sham-Controlled Clinical Trial. |  |  |  |  |  |  |
| Sandoval-Salinas et al. | Are Radial Pressure Waves Effective for the Treatment of Moderate or Mild to Moderate Erectile Dysfunction? A Randomized Sham Therapy Controlled Clinical Trial |  |  |  |  |  |  |
| Mykoniatis et al. | The Effect of Combination Treatment With Low-Intensity Shockwave Therapy and Tadalafil on Mild and Mild-To-Moderate Erectile Dysfunction: A Double-Blind, Randomized, Placebo-Controlled Clinical Trial |  |  |  |  |  |  |
| Kitrey et al. | Penile Low Intensity Shock Wave Treatment is Able to Shift PDE5i Non responders to Responders: A Double-Blind, Sham Controlled Study |  |  |  |  |  |  |
| Setiawan et al. | An update in improving erectile dysfunction therapy in Indonesia by using Li-ESWT and tadalafil combination — vascular endothelial growth factor and peak systolic velocity comparison: a randomized clinical trial |  |  |  |  |  |  |

*Domains* - D1: Randomisation process; D2: Deviations from the intended interventions; D3: Missing outcome data; D4 Measurement of the outcome; D5 selection of the reported result.

*Judgement*: Low Risk – Some concerns – High Risk

*Legend*: PDE5i: phosphodiesterase type 5 inhibitors; Li-ESWT: Low Intensity External Shock Wave Therapy; IIEF-5: International Index of Erectile Function Questionnaire – 5.

| Author | Title | D1 | D2 | D3 | D4 | D5 | Overall |
| --- | --- | --- | --- | --- | --- | --- | --- |
| Gallo et al. | Adjuvant daily therapy with L-arginine 2,500 mg and tadalafil 5 mg increases efficacy and duration of benefits of low-intensity extracorporeal shock wave therapy for erectile dysfunction: A prospective, randomized, single-blinded study with 1-year follow-up |  |  |  |  |  |  |
| Sandoval-Salinas et al. | Are Radial Pressure Waves Effective for the Treatment of Moderate or Mild to Moderate Erectile Dysfunction? A Randomized Sham Therapy Controlled Clinical Trial |  |  |  |  |  |  |
| Kitrey et al. | Penile Low Intensity Shock Wave Treatment is Able to Shift PDE5i Non responders to Responders: A Double-Blind, Sham Controlled Study |  |  |  |  |  |  |
| Setiawan et al. | An update in improving erectile dysfunction therapy in Indonesia by using Li-ESWT and tadalafil combination — vascular endothelial growth factor and peak systolic velocity comparison: a randomized clinical trial |  |  |  |  |  |  |

**Supplementary Table 2B**. Risk of bias for the study regarding the erectile function measured with EHS, at baseline vs at the end of follow up, according to the RoB-II tool

*Domains* - D1: Randomisation process; D2: Deviations from the intended interventions; D3: Missing outcome data; D4 Measurement of the outcome; D5 selection of the reported result.

*Judgement*: Low Risk – Some concerns – High Risk

*Legend*: PDE5i: phosphodiesterase type 5 inhibitors; Li-ESWT: Low Intensity External Shock Wave Therapy; EHS: Erection Hardness Score.

| Author | Title | D1 | D2 | D3 | D4 | D5 | D6 | D7 | Overall |
| --- | --- | --- | --- | --- | --- | --- | --- | --- | --- |
| Verze et al. | Efficacy and safety of low-intensity shockwave therapy plus tadalafil 5 mg once daily in men with type 2 diabetes mellitus and erectile dysfunction: a matched-pair comparison study |  |  |  |  |  |  |  |  |

**Supplementary Table 3.** Risk of bias for the study regarding the erectile function measured with IIEF-5, at baseline vs at the end of follow up, according to the Robins-I tool.

*Domains* - D1: Bias due to counfounding; D2: Bias due to selection of participants; D3: Bias in classification of interventions; D4: Bias due to deviations from intended interventions; D5: Bias to missing data; D6: Bias in measurement of outcomes; D7: Bias in selection of the reported result.

*Judgement*: Low – Moderate – Serious – Critical

*Legend: IIEF-5: International Index of Erectile Function Questionnaire – 5*
