# Supplementary material for: Comparative efficacy of combination therapy including regenerative therapies versus monotherapy for erectile dysfunction: A systematic review and meta‐analysis
Source: Andrology. 2025 Aug 12;14(2):358–67. doi: 10.1111/andr.70108 (PMC12842854; doi:10.1111/andr.70108)
Supplement: Supplementary file 2 — Supporting Information [file ANDR-14-358-s002.docx]

**Supplementary Figure 1: Forest plot comparing baseline IIEF-5 scores between combination and monotherapy at baseline, stratified by the type of monotherapy (PDE5i or Li-ESWT)**


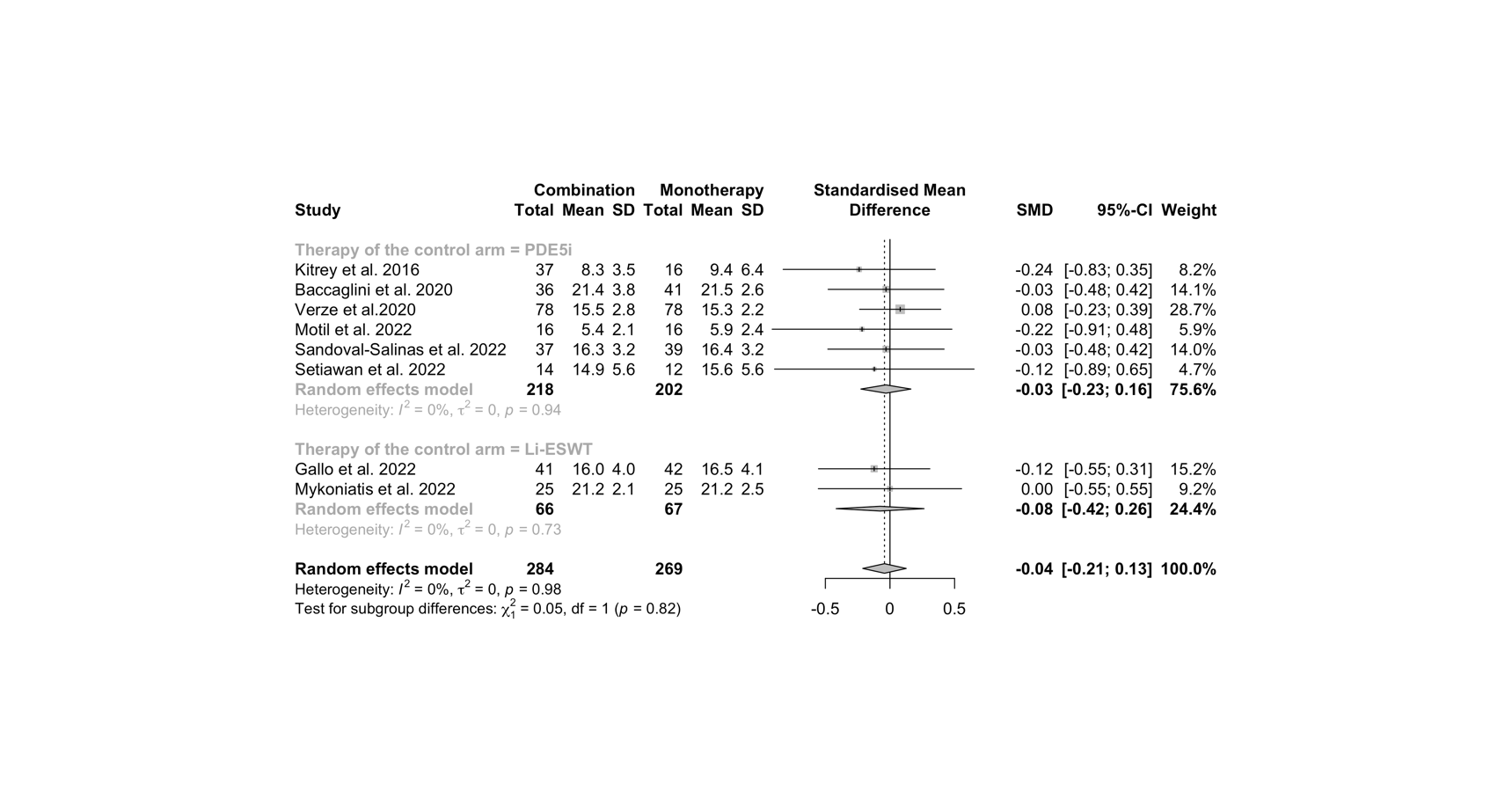


*Legend -* PDE5i: phosphodiesterase type 5 inhibitors; Li-ESWT: Low Intensity External Shock Wave Therapy; IIEF-5: International Index of Erectile Function Questionnaire – 5; SD: standard deviation; SMD: standard mean difference; 95%-CI: 95% Confidence Interval.

**Supplementary Figure 2: Forest plot of post treatment EHS scores in the combination vs monotherapy groups, stratified by the type of monotherapy (PDE5i or Li-ESWT)**

**
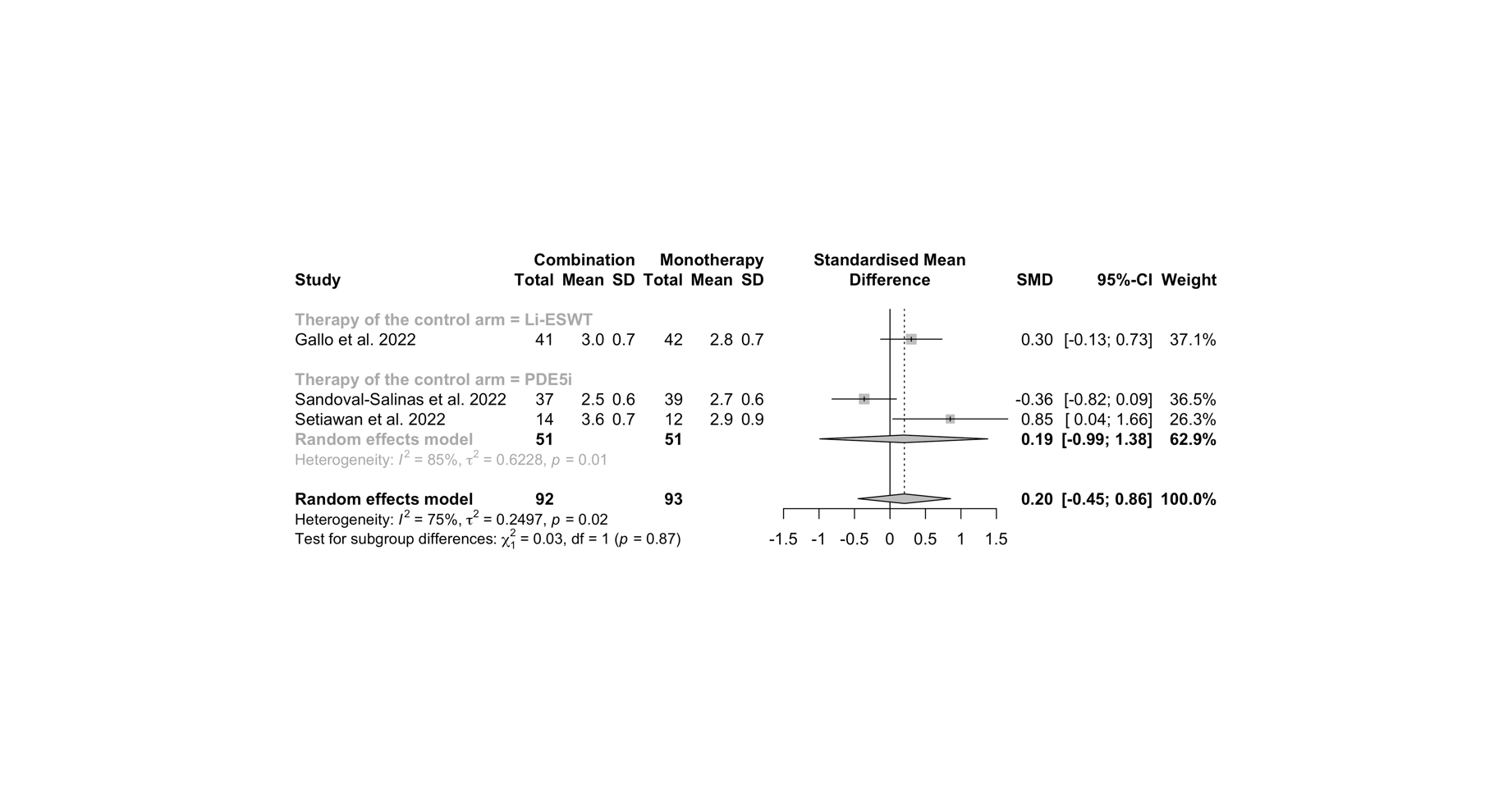
**

*Legend -* PDE5i: phosphodiesterase type 5 inhibitors; Li-ESWT: Low Intensity External Shock Wave Therapy; EHS: Erection Hardness Score; SD: standard deviation; SMD: standard mean difference; 95%-CI: 95% Confidence Interval.

**Supplementary Figure 3: Forest plot of IIEF-5 scores in the combination vs monotherapy groups at 6 months follow-up date, stratified by the type of monotherapy (PDE5i or Li-ESWT)**

**
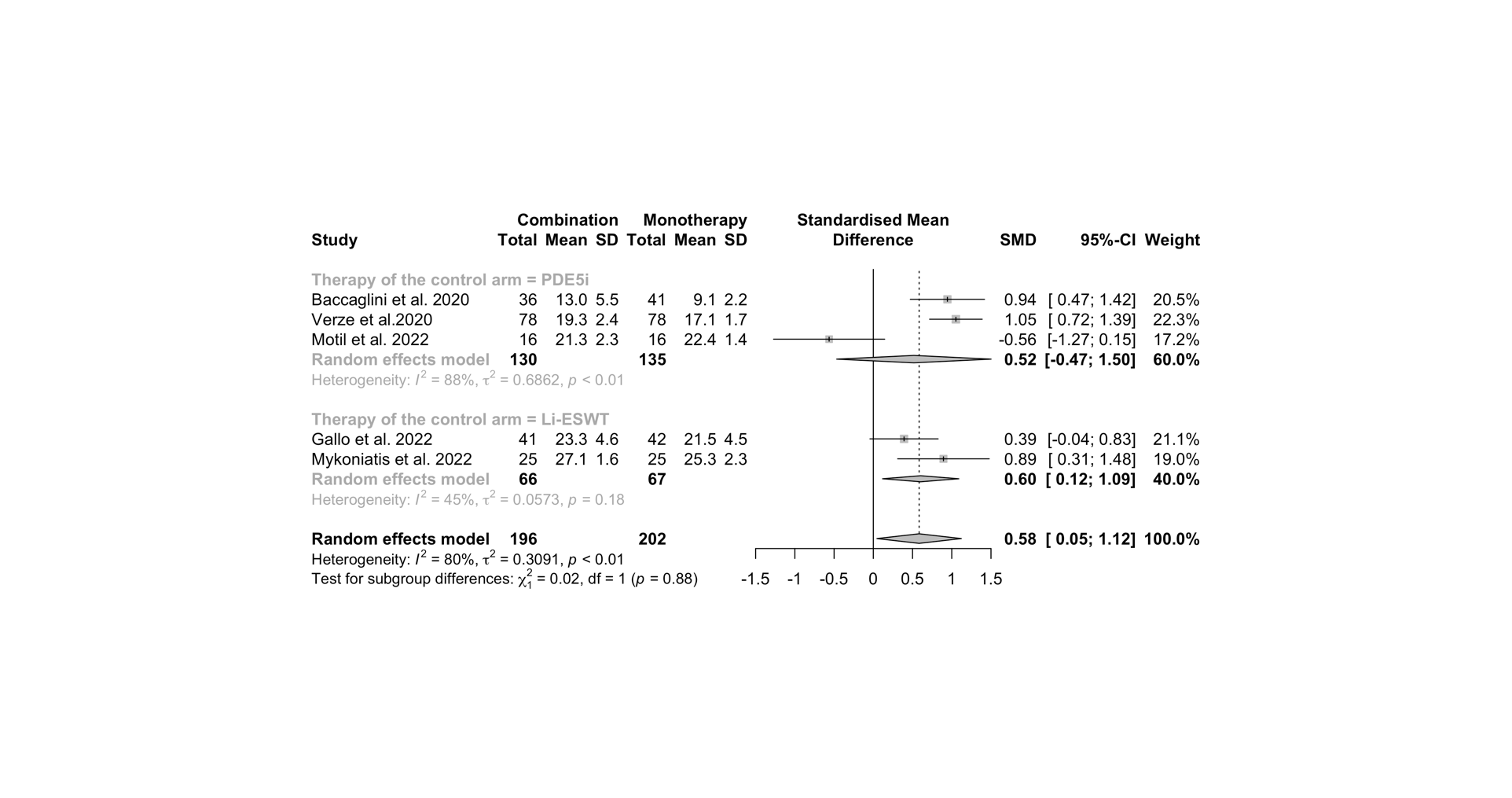
**

*Legend -* PDE5i: phosphodiesterase type 5 inhibitors; Li-ESWT: Low Intensity External Shock Wave Therapy; IIEF-5: International Index of Erectile Function Questionnaire – 5; SD: standard deviation; SMD: standard mean difference; 95%-CI: 95% Confidence Interval.

**Supplementary Figure 4: Forest plot of the difference in post treatment IIEF-5 scores compared to baseline in the monotherapy (A) and combination (B) groups, after exclusion of Baccaglini et al. 2020**

| **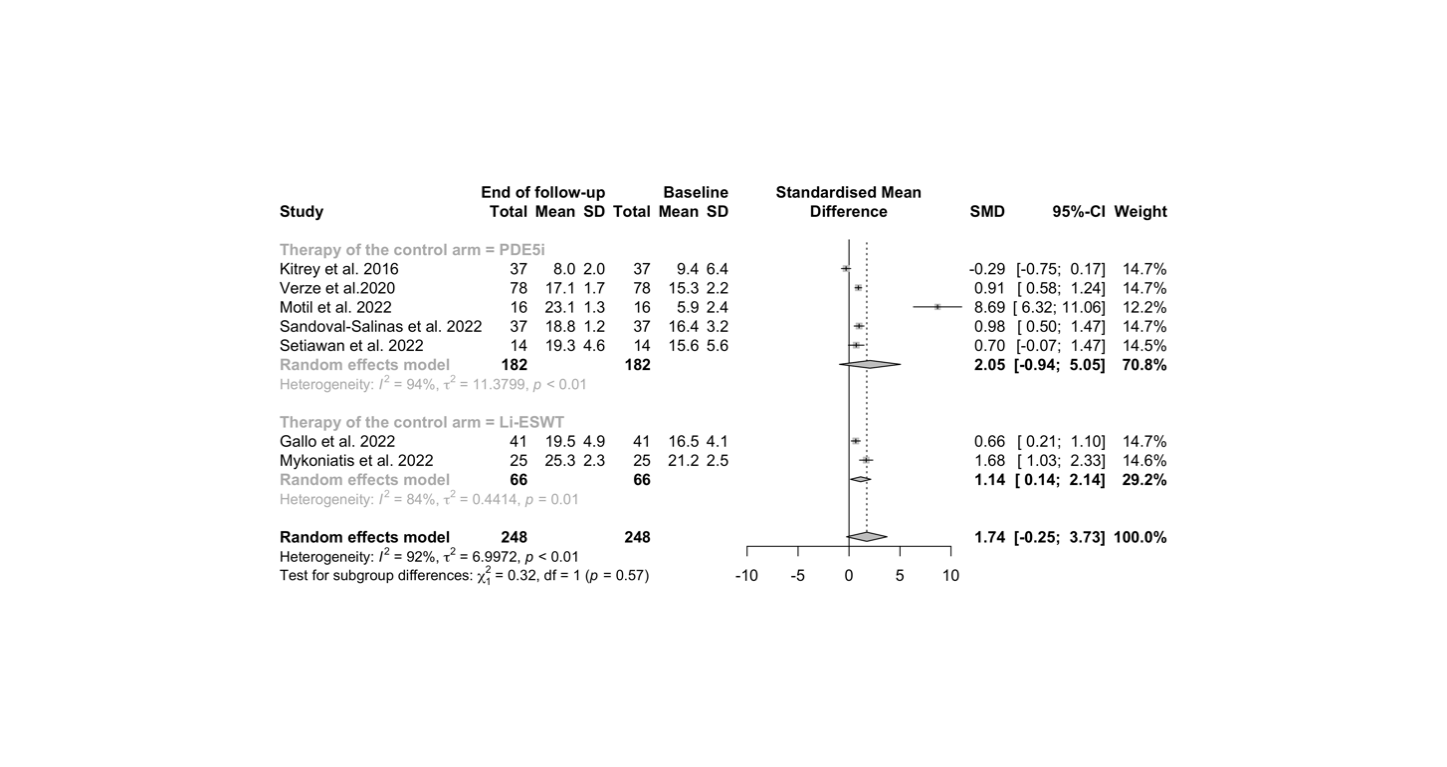A)** | **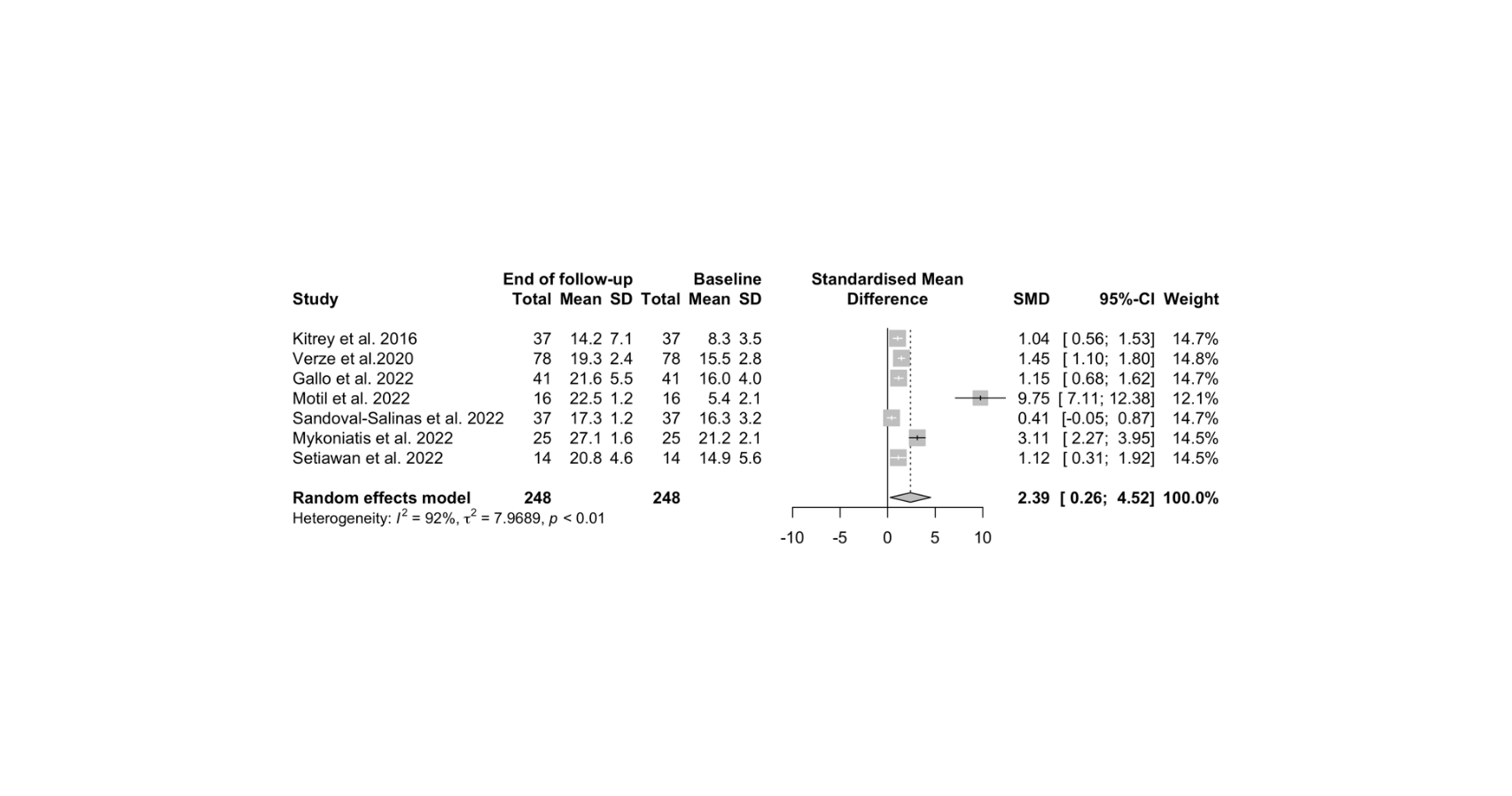**  **B)** |
| --- | --- |

*Legend -* PDE5i: phosphodiesterase type 5 inhibitors; Li-ESWT: Low Intensity External Shock Wave Therapy; IIEF-5: International Index of Erectile Function Questionnaire – 5; SD: standard deviation; SMD: standard mean difference; 95%CI: 95% Confidence Interval. **Supplementary Figure 5: Forest plot of the difference in post treatment EHS scores compared to baseline scores in the monotherapy (A) and combination (B) groups**

| **A)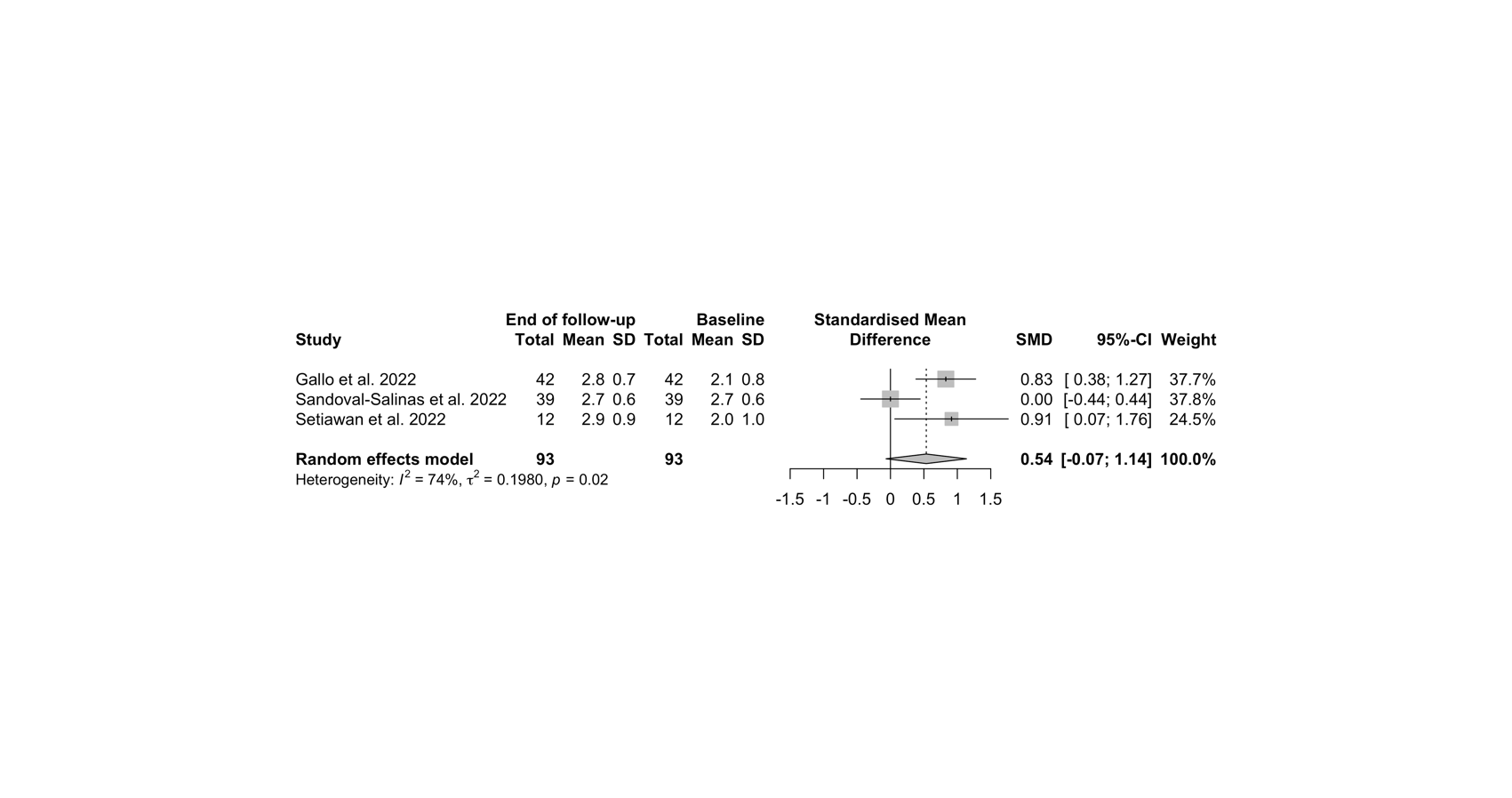** | **B)**  **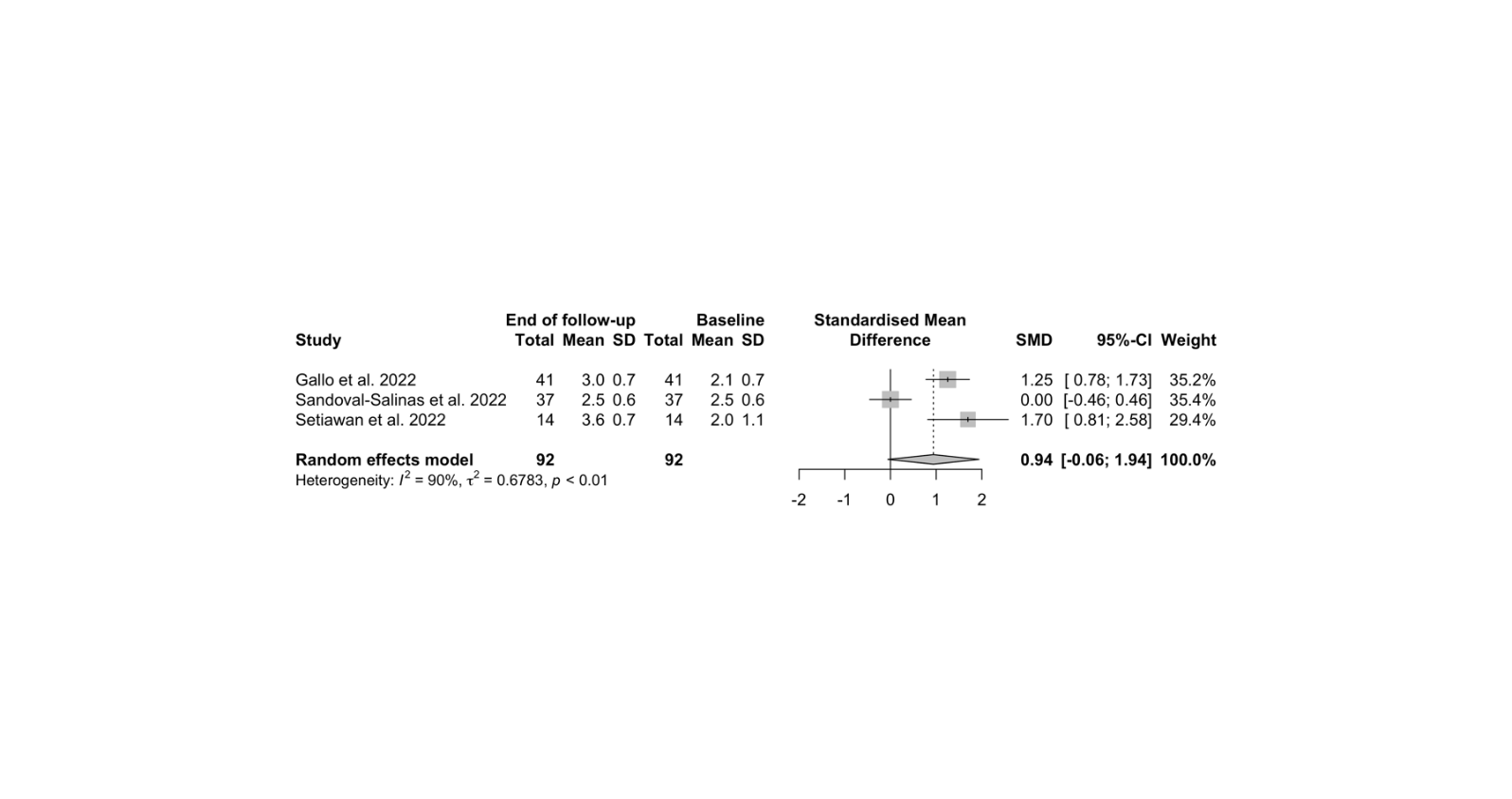** |
| --- | --- |

*Legend* - EHS: Erection Hardness Score; SD: standard deviation; SMD: standard mean difference; 95%-CI: 95% Confidence Interval.

**Supplementary Figure 6: Funnel plots for publication bias across for the different outcomes, A) Post-treatment IIEF-5 combination vs monotherapy groups; B) Baseline IIEF-5 combination vs monotherapy groups; C) Post-treatment vs baseline IIEF-5 in the monotherapy group; D) Post-treatment vs baseline IIEF-5 in the combination group.**

| **A)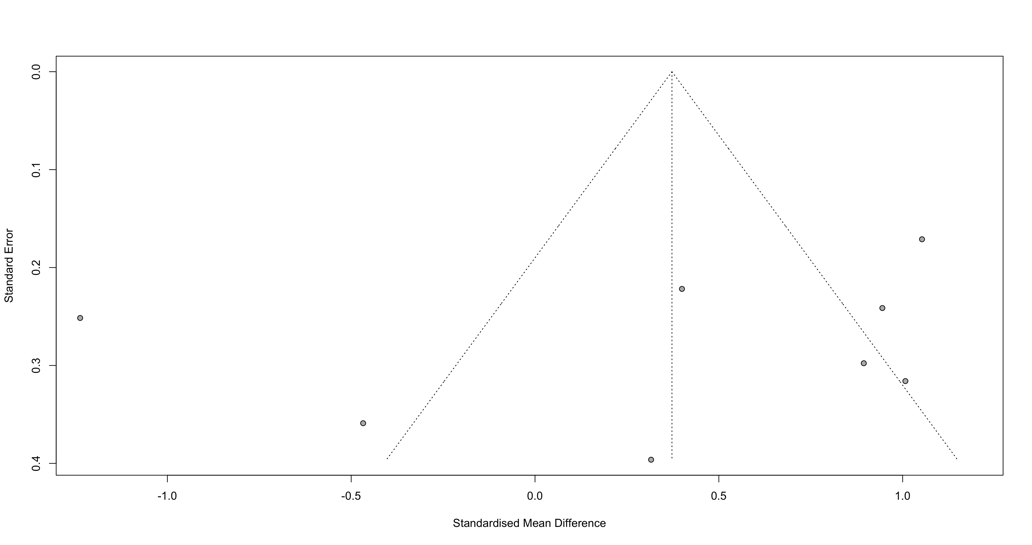** | **B)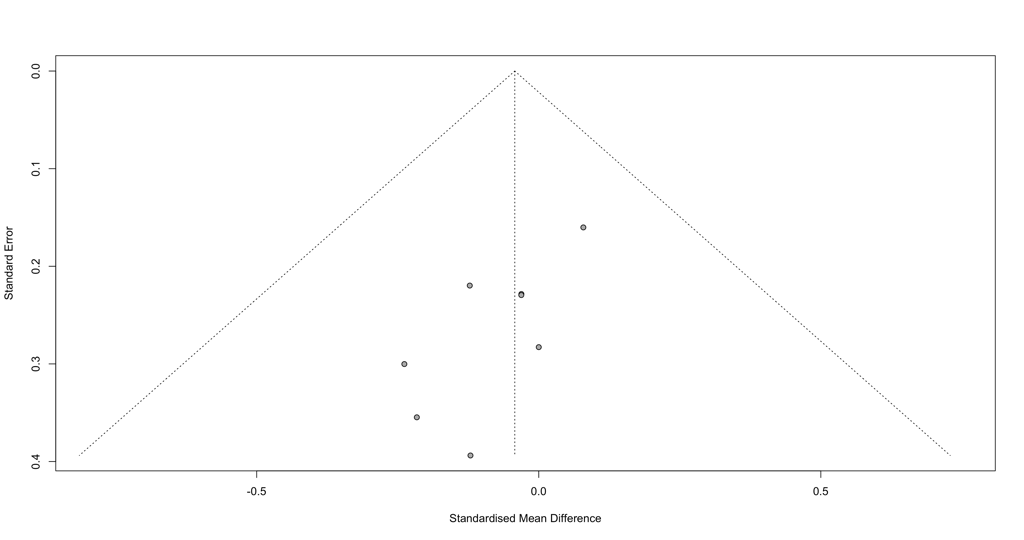** |
| --- | --- |
| **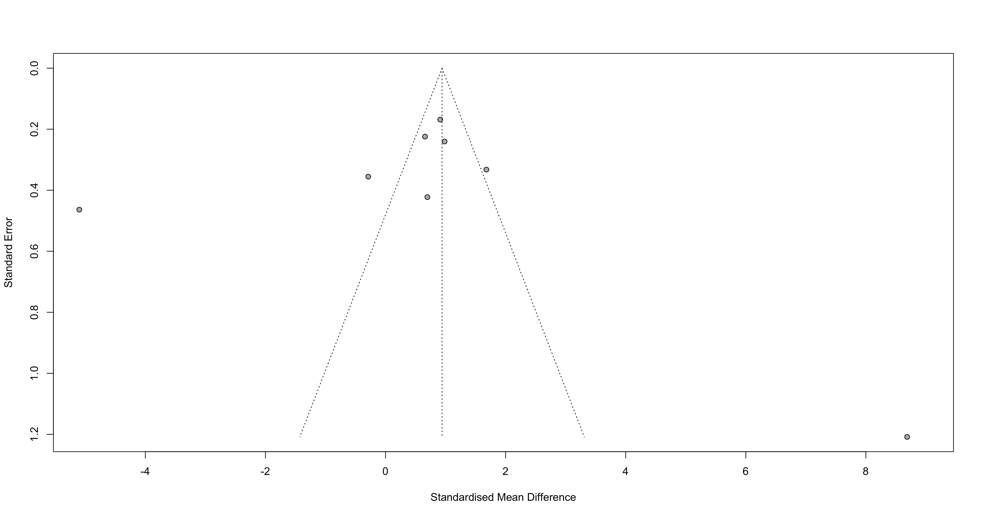C)** | **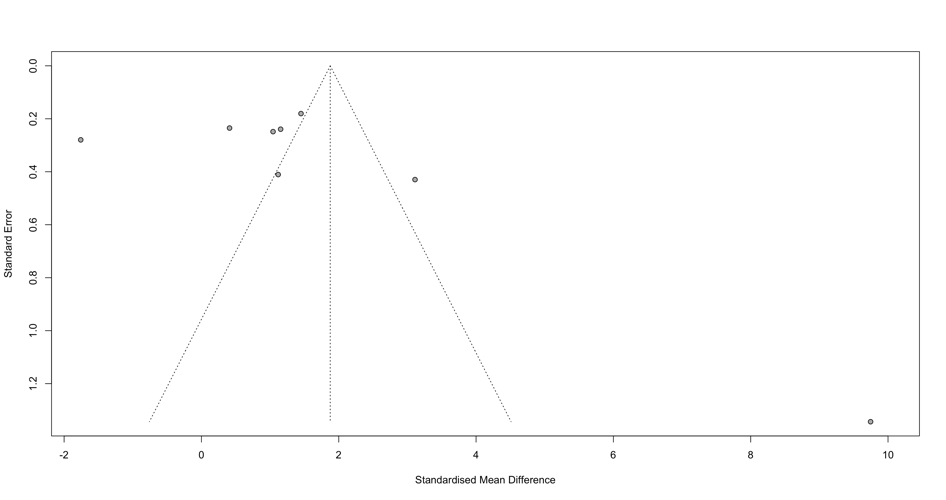D)** |

*Legend* - IIEF-5: International Index of Erectile Function Questionnaire

| Supplementary Table 1. Characteristics of the Li-ESWT treatment in all included studies | | |
| --- | --- | --- |
| Author; year of publication | **Title** | **Schedule** |
| Gallo et al. 2022 | Adjuvant daily therapy with L-arginine 2,500 mg and tadalafil 5 mg increases efficacy and duration of benefits of low-intensity extracorporeal shock wave therapy for erectile dysfunction: A prospective, randomized, single-blinded study with 1-year follow-up | 1 weekly application for 6 weeks. Each session consisted of 3000 SW |
| Motil et al. 2022 | Linear Low-Intensity Extracorporeal Shockwave Therapy as a Method for Penile Rehabilitation in Erectile Dysfunction Patients after Radical Prostatectomy: A Randomized, Single-Blinded, Sham-Controlled Clinical Trial. | 1 weekly application for 4 weeks. Each session consisted of 4000 SW |
| Baccaglini et al. 2020 | The Role of the Low-Intensity Extracorporeal Shockwave Therapy on Penile Rehabilitation After Radical Prostatectomy: A Randomized Clinical Trial | 1 weekly application for 8 weeks. |
| Sandoval-salinas et al. 2022 | Are Radial Pressure Waves Effective for the Treatment of Moderate or Mild to Moderate Erectile Dysfunction? A Randomized Sham Therapy Controlled Clinical Trial | 1 weekly application for 6 weeks. Each session consisted of 4000 SW in the body of the penis and 2000 SW the perineal area. |
| Verze et al. 2020 | Efficacy and safety of low-intensity shockwave therapy plus tadalafil 5 mg once daily in men with type 2 diabetes mellitus and erectile dysfunction: a matched-pair comparison study | 2 weekly applications for 3 weeks. Each session consisted of 1500 to 2400 SW. |
| Mykoniatis et al. 2022 | The Effect of Combination Treatment With Low-Intensity Shockwave Therapy and Tadalafil on Mild and Mild-To-Moderate Erectile Dysfunction: A Double-Blind, Randomized, Placebo-Controlled Clinical Trial | 2 weekly applications for 3 weeks. Each session consisted of 5000 SW. |
| Kitrey et al. 2016 | Penile Low Intensity Shock Wave Treatment is Able to Shift PDE5i Non responders to Responders: A Double-Blind, Sham Controlled Study | 2 weekly applications for 3 weeks. Each session consisted of 1500 SW. |
| Setiawan et al. 2022 | An update in improving erectile dysfunction therapy in Indonesia by using Li-ESWT and tadalafil combination — vascular endothelial growth factor and peak systolic velocity comparison: a randomized clinical trial | 2 weekly applications for 4 weeks. Each session consisted of 1500 SW. |

*Legend* – SW: Shock waves; Li-ESWT: Low Intensity External Shock Wave Therapy; PDE5i: phosphodiesterase type 5 inhibitors.

| Supplementary Table 2A. Risk of bias for the study regarding the erectile function measured with IIEF-5, at the end of follow up vs baseline, according to the RoB 2 tool | | | | | | | |
| --- | --- | --- | --- | --- | --- | --- | --- |
| Author | **Title** | **D1** | **D2** | **D3** | **D4** | **D5** | **Overall** |
| Baccaglini et al. | The Role of the Low-Intensity Extracorporeal Shockwave Therapy on Penile Rehabilitation After Radical Prostatectomy: A Randomized Clinical Trial |  |  |  |  |  |  |
| Gallo et al. | Adjuvant daily therapy with L-arginine 2,500 mg and tadalafil 5 mg increases efficacy and duration of benefits of low-intensity extracorporeal shock wave therapy for erectile dysfunction: A prospective, randomized, single-blinded study with 1-year follow-up |  |  |  |  |  |  |
| Motil et al. | Linear Low-Intensity Extracorporeal Shockwave Therapy as a Method for Penile Rehabilitation in Erectile Dysfunction Patients after Radical Prostatectomy: A Randomized, Single-Blinded, Sham-Controlled Clinical Trial. |  |  |  |  |  |  |
| Sandoval-Salinas et al. | Are Radial Pressure Waves Effective for the Treatment of Moderate or Mild to Moderate Erectile Dysfunction? A Randomized Sham Therapy Controlled Clinical Trial |  |  |  |  |  |  |
| Mykoniatis et al. | The Effect of Combination Treatment With Low-Intensity Shockwave Therapy and Tadalafil on Mild and Mild-To-Moderate Erectile Dysfunction: A Double-Blind, Randomized, Placebo-Controlled Clinical Trial |  |  |  |  |  |  |
| Kitrey et al. | Penile Low Intensity Shock Wave Treatment is Able to Shift PDE5i Non responders to Responders: A Double-Blind, Sham Controlled Study |  |  |  |  |  |  |
| Setiawan et al. | An update in improving erectile dysfunction therapy in Indonesia by using Li-ESWT and tadalafil combination — vascular endothelial growth factor and peak systolic velocity comparison: a randomized clinical trial |  |  |  |  |  |  |

*Domains* - D1: Randomisation process; D2: Deviations from the intended interventions; D3: Missing outcome data; D4 Measurement of the outcome; D5 selection of the reported result.

*Judgement*: Low Risk – Some concerns – High Risk

*Legend*: PDE5i: phosphodiesterase type 5 inhibitors; Li-ESWT: Low Intensity External Shock Wave Therapy; IIEF-5: International Index of Erectile Function Questionnaire – 5.

| Supplementary Table 2B. Risk of bias for the study regarding the erectile function measured with EHS, at the end of follow up vs baseline, according to the RoB 2 tool | | | | | | | |
| --- | --- | --- | --- | --- | --- | --- | --- |
| Author | **Title** | **D1** | **D2** | **D3** | **D4** | **D5** | **Overall** |
| Gallo et al. | Adjuvant daily therapy with L-arginine 2,500 mg and tadalafil 5 mg increases efficacy and duration of benefits of low-intensity extracorporeal shock wave therapy for erectile dysfunction: A prospective, randomized, single-blinded study with 1-year follow-up |  |  |  |  |  |  |
| Sandoval-Salinas et al. | Are Radial Pressure Waves Effective for the Treatment of Moderate or Mild to Moderate Erectile Dysfunction? A Randomized Sham Therapy Controlled Clinical Trial |  |  |  |  |  |  |
| Kitrey et al. | Penile Low Intensity Shock Wave Treatment is Able to Shift PDE5i Non responders to Responders: A Double-Blind, Sham Controlled Study |  |  |  |  |  |  |
| Setiawan et al. | An update in improving erectile dysfunction therapy in Indonesia by using Li-ESWT and tadalafil combination — vascular endothelial growth factor and peak systolic velocity comparison: a randomized clinical trial |  |  |  |  |  |  |

*Domains* - D1: Randomisation process; D2: Deviations from the intended interventions; D3: Missing outcome data; D4 Measurement of the outcome; D5 selection of the reported result.

*Judgement*: Low Risk – Some concerns – High Risk

*Legend*: PDE5i: phosphodiesterase type 5 inhibitors; Li-ESWT: Low Intensity External Shock Wave Therapy; EHS: Erection Hardness Score.

| Supplementary Table 3. Risk of bias for the study regarding the erectile function measured with IIEF-5, at baseline vs at the end of follow up, according to the Robins-I tool. | | | | | | | | | |
| --- | --- | --- | --- | --- | --- | --- | --- | --- | --- |
| Author | **Title** | **D1** | **D2** | **D3** | **D4** | **D5** | **D6** | **D7** | **Overall** |
| Verze et al. | Efficacy and safety of low-intensity shockwave therapy plus tadalafil 5 mg once daily in men with type 2 diabetes mellitus and erectile dysfunction: a matched-pair comparison study |  |  |  |  |  |  |  |  |

*Domains* - D1: Bias due to counfounding; D2: Bias due to selection of participants; D3: Bias in classification of interventions; D4: Bias due to deviations from intended interventions; D5: Bias to missing data; D6: Bias in measurement of outcomes; D7: Bias in selection of the reported result.

*Judgement*: Low – Moderate – Serious – Critical

*Legend: IIEF-5: International Index of Erectile Function Questionnaire – 5*
